# Optimal selection of specimens for metagenomic next-generation sequencing in diagnosing periprosthetic joint infections

**DOI:** 10.3389/fcimb.2024.1356804

**Published:** 2024-03-04

**Authors:** Jun Tan, Lingxiao Wu, Lijuan Zhan, Minkui Sheng, Zhongxin Tang, Jianzhong Xu, Haijun Ma

**Affiliations:** ^1^ Department of Mini-invasive Spinal Surgery, The Third People’s Hospital of Henan Province, Zhengzhou, Henan, China; ^2^ Department of Orthopedic Surgery, The First Affiliated Hospital of Zhengzhou University, Zhengzhou, Henan, China; ^3^ Department of Neurology, People’s Hospital of Zhengzhou, Zhengzhou, Henan, China

**Keywords:** metagenomic, prosthesis-related infections, next-generation sequencing - NGS, diagnosis, sonication

## Abstract

**Objective:**

This study aimed to assess the diagnostic value of metagenomic next-generation sequencing (mNGS) across synovial fluid, prosthetic sonicate fluid, and periprosthetic tissues among patients with periprosthetic joint infection (PJI), intending to optimize specimen selection for mNGS in these patients.

**Methods:**

This prospective study involved 61 patients undergoing revision arthroplasty between September 2021 and September 2022 at the First Affiliated Hospital of Zhengzhou University. Among them, 43 cases were diagnosed as PJI, and 18 as aseptic loosening (AL) based on the American Musculoskeletal Infection Society (MSIS) criteria. Preoperative or intraoperative synovial fluid, periprosthetic tissues, and prosthetic sonicate fluid were collected, each divided into two portions for mNGS and culture. Comparative analyses were conducted between the microbiological results and diagnostic efficacy derived from mNGS and culture tests. Furthermore, the variability in mNGS diagnostic efficacy for PJI across different specimen types was assessed.

**Results:**

The sensitivity and specificity of mNGS diagnosis was 93% and 94.4% for all types of PJI specimens; the sensitivity and specificity of culture diagnosis was 72.1% and 100%, respectively. The diagnostic sensitivity of mNGS was significantly higher than that of culture (X^2^ = 6.541, *P*=0.011), with no statistically significant difference in specificity (X^2^ = 1.029, *P*=0.310). The sensitivity of the synovial fluid was 83.7% and the specificity was 94.4%; the sensitivity of the prosthetic sonicate fluid was 90.7% and the specificity was 94.4%; and the sensitivity of the periprosthetic tissue was 81.4% and the specificity was 100%. Notably, the mNGS of prosthetic sonicate fluid displayed a superior pathogen detection rate compared to other specimen types.

**Conclusion:**

mNGS can function as a precise diagnostic tool for identifying pathogens in PJI patients using three types of specimens. Due to its superior ability in pathogen identification, prosthetic sonicate fluid can replace synovial fluid and periprosthetic tissue as the optimal sample choice for mNGS.

## 1 Introduction

Periprosthetic joint infection (PJI) stands as one of the most catastrophic and challenging complications following total joint arthroplasty (TJA) (Gallo, 2020; Rietbergen et al., 2016). Rapid and accurate identification of pathogenic bacteria is crucial for determining the appropriate surgical approach and for selecting effective antibacterial drugs (Hersh et al., 2019; Young et al., 2023). Although widely used for diagnosing PJI, traditional blood tests like C-reactive protein (CRP), erythrocyte sedimentation rate (ESR), and white blood cell counts (WBC) struggle to identify the causative organism (Nodzo et al., 2015). Microbial culture is extensively employed for diagnosing the pathogenesis of PJI; however, it exhibits a high false-negative rate. Approximately 40% of clinically diagnosed PJI cases yield negative culture results due to the existence of bacterial biofilms and prior antibiotic treatment (Rak et al., 2013; Yoon et al., 2017; Malekzadeh et al., 2010). Molecular diagnostic techniques based on polymerase chain reaction (PCR) are extensively employed in detecting pathogenic microorganisms (Hartley and Harris, 2014). However, multiplex PCR techniques exhibit low diagnostic sensitivity, and 16S rDNA/rRNA PCR encounters difficulty in identifying fungal or multi-pathogenic infections, displaying a poor ability in identifying contaminating bacteria (Villa et al., 2017; Huang et al., 2018).

Next-generation sequencing, particularly metagenomic next-generation sequencing (mNGS), is a swiftly developing and extensively employed technology in the clinical diagnosis of pathogenic microorganisms (Chiu and Miller, 2019). mNGS integrates high-throughput sequencing and bioinformatics analysis to discern sequence data from all nucleic acid fragments within samples, detecting microbial species and their respective abundances relying on the BLAST database (Gu et al., 2021). Wilson et al. (2014) initially utilized mNGS for clinically diagnosing neuroleptospirosis in a 14-year-old patient experiencing severe meningoencephalitis. The successful diagnosis enabled targeted antibiotic treatment, leading to the patient’s eventual recovery and demonstrating, for the first time, the utility of mNGS in clinical diagnosis (Wilson et al., 2014). The progressive application of mNGS in diagnosing infectious diseases has demonstrated enhanced detection of pathogenic microorganisms, particularly in conditions like ocular, intracranial, and pulmonary infections (O’Flaherty et al., 2018; Brown et al., 2018; Doan et al., 2017). However, there is a scarcity of studies utilizing mNGS for detecting pathogenic microorganisms in PJI, primarily relying on a single specimen for pathogen detection, such as synovial fluid, prosthetic sonicate fluid, and periprosthetic tissue (Dudareva et al., 2018). mNGS results correlate with the tissue type, volume of the sampled specimen, and the proportions of human DNA and pathogenic bacterial DNA in the sample (Thoendel et al., 2016). Variations exist in mNGS test outcomes among distinct samples obtained from the same patient (Miao et al., 2018). Choosing the most suitable specimen is crucial for pathogen identification, not just for traditional culture methods but also for molecular diagnostic technologies (Huang et al., 2018; Larsen et al., 2018; Flurin et al., 2022).

This study aimed to prospectively assess the diagnostic efficacy of mNGS across three different specimen types obtained from patients with PJI. This approach should facilitate the selection of more suitable tests for detecting pathogens in these patients by employing multiple samples.

## 2 Materials and methods

### 2.1 Patient selection

This study received approval from our institution’s Medical Ethics Committee (Approval No:2020-KY-821), and all patients provided informed consent by signing an informed consent form. We prospectively included 61 patients who underwent hip or knee revision procedures at our institution from September 2021 to September 2022 due to PJI or aseptic loosening (AL).

The diagnosis of PJI after TJA is based primarily on the American Musculoskeletal Infection Association (MSIS) criteria, confirmed by meeting one major criterion or 4 out of the 6 minor criteria (Parvizi et al., 2011). The major criteria include (1): Isolation of the same pathogen from two distinct tissue or fluid specimens sourced from infected joints (2); Presence of a sinus tract communicating with the prosthesis. Minor criteria consist of (1): Elevated ESR and CRP levels (2); Increased synovial fluid WBC (3); Elevated synovial fluid neutrophil percentage (4); Presence of pus in the affected joint (5); Isolation of a microorganism in either periprosthetic tissue or fluid culture (6); The intraoperative microscopic examination of frozen sections of infected tissue revealed an observation of greater than 5 neutrophils in all 5 high-magnification fields.

The inclusion criteria comprised (1): Patients diagnosed with PJI or AL according to the MSIS diagnostic criteria, undergoing revision surgery at our institution (2); Patients undergoing mNGS testing and microbial culture analysis of synovial fluid, prosthetic sonication fluid from periprosthetic tissue, and periprosthetic tissue (3); Patients with complete medical records. The exclusion criteria were (1): Patients with samples suspected of contamination during collection, transportation, or processing (2); Patients with an unclear diagnosis or incomplete clinical information (3); Patients presenting other inflammatory lesions or malignancies potentially affecting the outcomes.

### 2.2 Specimen collection

The same surgeon conducted arthrocentesis to collect synovial fluid under ultrasound guidance either before or during the operation.

Ultrasound cleavage of the prosthesis removed intraoperatively was performed according to the method of Trampuz et al. (2007) for reference. The process included placing the extracted implants in a sterile, sealable container, submerging them in 500 ml of Ringer’s solution, subjecting the samples to 30 seconds of vortexing and oscillation, and subsequently performing sonication for five min (40 ± 2 kHz, 0.22 ± 0.04 W/cm^2^) in a sterile water bath. Subsequently, after vortexing and oscillation for an additional 30 seconds, the sonicated product was transferred to a centrifuge tube and centrifuged at 4000 rpm/min for 15 minutes. The supernatant was then removed to collect the prosthetic sonicated fluid.

Intraoperatively, tissue samples were collected from five different sites with the most obvious inflammation, subsequently sheared, ground, lysed, and centrifuged to produce tissue homogenates for further analysis.

The three types of specimens obtained as described earlier were split into two portions: one for culture, while the other was frozen at -80°C for mNGS.

### 2.3 Microbial culture

Synovial fluid and prosthetic sonication fluid direct smears underwent acid-fast staining and were then cultured aerobically and anaerobically (35-37°C, 5%-7% CO_2_) on blood agar plates for 6 and 14 days, respectively. The residual synovial fluid and prosthetic sonication fluid were introduced into BACTEC Peds Plus/F bottles and then incubated for 5 days in a BACTEC 9240 instrument (Becton Dickinson, Cockeysville, MD, USA). Positive blood culture bottles were Gram stained and then inoculated. Periprosthetic tissue homogenates were cultured on blood agar plates and incubated aerobically, anaerobically, and for fungal growth for 14 days.

Species identification and drug sensitivity analyses were conducted utilizing the VITEK 2-Compact (bioMérieux, Lyon, France), a fully automated system for bacterial identification and drug sensitivity analysis.

### 2.4 Metagenomic next-generation sequencing

#### 2.4.1 DNA extraction

Total genomic DNA was extracted from samples using the TIANamp Micro DNA Kit (DP316, TIANamp Biotech, Beijing, China) according to the manufacturer’s instructions. The extracted DNA underwent sonication using the Covaris S220 (Covaris, Woburn, MA, USA) to produce fragments ranging from 200 bp to 300 bp.

#### 2.4.2 DNA library preparation and sequencing

The construction of DNA library followed the protocol of the Nextera XT library construction Kit (Illumina, San Diego, CA, USA), where the extracted DNA was initially fragmented into ~300 bp fragments, followed by the addition of different index sequences. The library size and quantification were assessed using the Agilent 2100 bioanalyzer system (Agilent Technologies, CA, USA). Subsequent to this, accurate quantification was reaffirmed through qPCR (Bio-Rad CFX96, Hercules, CA, USA). Following the equal mixing of libraries, high-throughput sequencing was performed on the Illumina Nextseq 550 DX sequencing platform.

#### 2.4.3 Bioinformatics analysis

**Figure 1:**
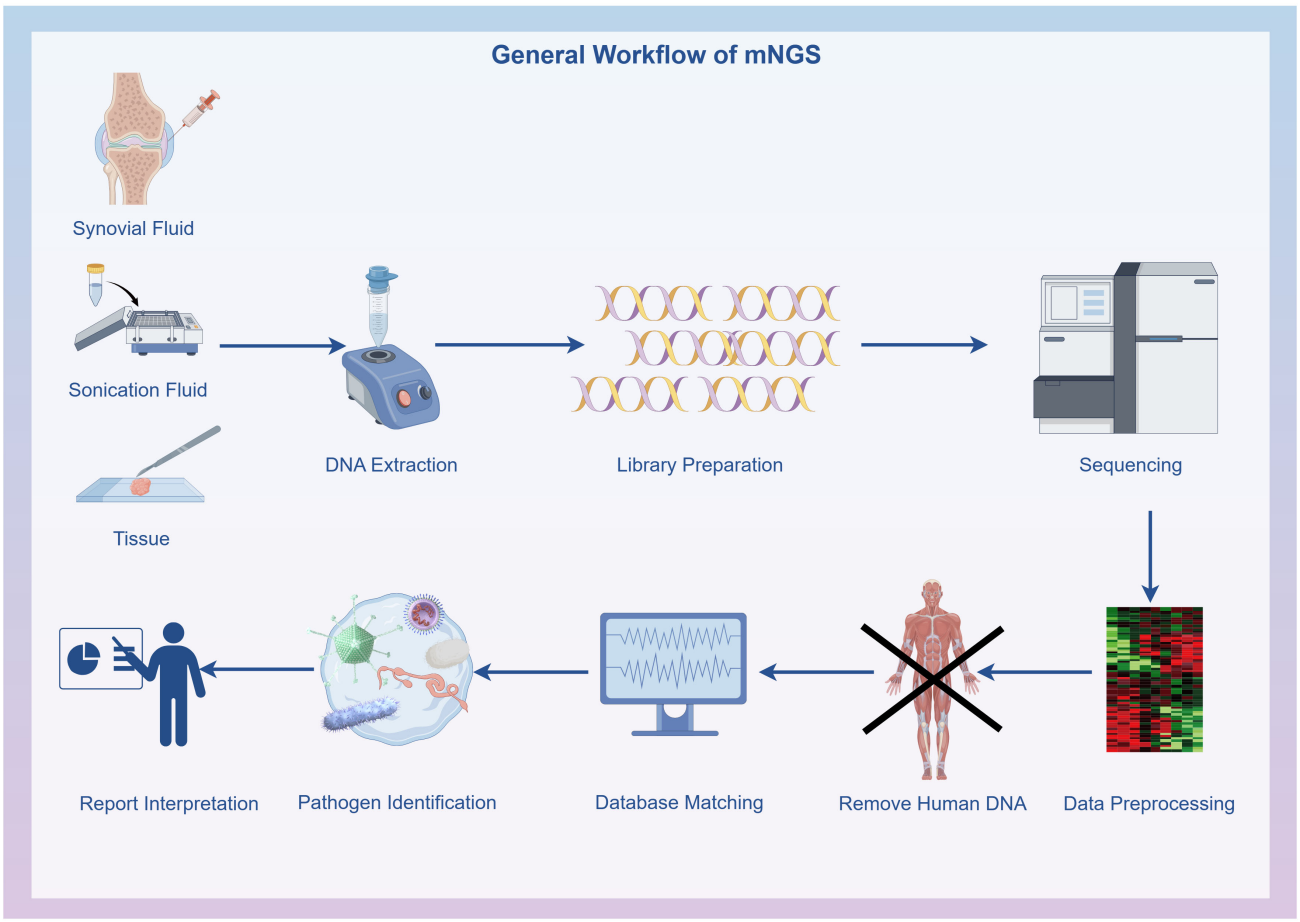
General workflow of metagenomic next-generation sequencing: Specimen collection, DNA extraction, DNA library preparation, Sequencing, Bioinformatics analysis and Result interpretation. The flowchart was drawn with Figdraw (www.figdraw.com).

Initial filtering of the raw data was conducted using FastQC software (version 0.11.7, http://bioinformatics.babraham.ac.uk/projects/fastqc/), including removing low-quality data, sequences shorter than 35 bp, repeated sequences, and adaptor contamination to produce high-quality sequencing data. Human host sequences were removed through mapping to the human reference genome (hg19) using the Burrows-Wheeler Alignment (version 0.7.13, http://bio-bwa.sourceforge.net) (Li and Durbin, 2009). The remaining data underwent analysis with Kraken2 (Wood et al., 2019) (version 2.1.1) and Bracken (Lu et al., 2017) (version 2.6.2) to identify and quantify pathogenic microorganisms. The resultant clinical test reports deliver accurate and reliable information on microbiological test results. The microbial reference whole-genome data were sourced from the National Center for Biotechnology Information (http://ncbi.nlm.nih.gov/genome) (**Figure 1**).

#### 2.4.4 Definition of the mNGS threshold

Relative abundance in genus level (RAG) refers to the proportion of matched microbial genera among all microorganisms of the same type (bacteria, fungi, viruses, parasites) detected. The genome coverage rate (CR) primarily represents the proportion of the detected pathogenic nucleic acid sequence length in relation to the total length of the reference genome used for comparison.

In this study, saline served as a negative control. mNGS were conducted concurrently for the same batch of samples (both the negative control and clinical samples). Microorganisms identified in negative controls are commonly viewed as environmental contaminants. If these microorganisms are detected in the same batch of clinical samples, they are generally classified as “background microorganisms” rather than “pathogenic microorganism”.

Referring to the proposal of Li et al. (2018) and considering our experimental findings, the thresholds were set as follows (1): As *Burkholderia, Aspergillus, Delftia, Sodaria, Sphingobium, Alternaria, Ralstonia, Albugo*, etc. can be detected in other types of samples from the same laboratory and are seldom associated with causing PJI (Zhang et al., 2019), we categorized them as background microorganisms. These microorganisms were recognized as causative pathogens only when RAG >80% (Ivy et al., 2018) (2). RAG >15% and the number of reads >100 were employed as the optimal thresholds for bacterial identification (3). Considering the limited quantity of nucleic acid obtained from a fungus, we established RAG >15% and the number of reads >50 as the optimal thresholds for fungi (Miao et al., 2018) (4). *Mycobacterium tuberculosis* was deemed positive when it met the criterion of having at least one read aligned to the reference genome at the species or genus level.

### 2.5 Statistical analysis

All clinical and laboratory data underwent analysis employing the non-parametric Mann-Whitney U test, chi-squared test, and independent-samples t-test. The sensitivities and specificities of microbiological tests were compared using the McNemar’s test to assess related proportions. Data analyses were conducted using SPSS 23.0 software (SPSS 23.0 for Windows, SPSS Inc., Chicago, IL, USA). *P* < 0.05 were deemed statistically significant.

## 3 Results

### 3.1 Patient characteristics

This study encompassed 61 patients, with 43 diagnosed with PJI and classified in the PJI group, while 18 patients diagnosed with AL were classified in the AL group based on the MSIS criteria. The PJI group comprised 43 patients (26 knees and 17 hips): 24 males, 19 females, with an average age of 62.31 ± 10.15 years, and a Body Mass Index (BMI) of 24.3 ± 2.9 kg/m^2^. There were 18 patients (11 knees and 7 hips) in the AL group, including 8 males and 10 females; with an average age of 63.57 ± 11.37 years, and BMI of 25.1 ± 2.15 kg/m^2^. There were no statistically significant differences in gender, age, or BMI between the two groups (*P* > 0.05). The characteristics of all the included patients are showed in **Table 1**.

**Table 1:** The characteristics of the PJI and AL groups.

Variable	Prosthetic joint infection (n = 43)	Aseptic failure (n = 18)	*P*
Sex, n (%)			0.417^*^
Male	24 (55.8%)	8 (44.4%)	
Female	19 (44.2%)	10 (55.6%)	
Age, yrs, mean (SD)	62.31 (10.15)	63.57 (11.37)	0.079^†^
BMI, kg/m^2^ (SD)	24.3 (2.91)	25.1 (2.15)	0.305^†^
Site of arthroplasty, n (%)			0.962^*^
Knee	26 (60.5%)	11 (61.1%)	
Hip	17 (39.5%)	7 (38.9%)	
Sinus tract, n (%)	4 (9.3%)	0 (0%)	0.012^*^
Antibiotics received two weeks prior to surgery, n (%)	27 (62.8%)	2 (11.1%)	<0.001^*^
Serum ESR, mm/h, mean (SD)	65.28 (38.51)	15.38 (12.07)	0.022^‡^
Serum CRP, mg/dl, mean (SD)	45.57 (30.37)	4.03 (1.38)	0.015^‡^
Synovial WBC, cells/ml mean (SD)	17931 (8237.07)	612.91 (533.76)	0.004^‡^
Synovial PMN, % (SD)	73.55 (16.74)	38.27 (13.58)	0.031^‡^

^†^Independent-samples t-test. ^*^Chi-squared test. ^‡^Mann-Whitney U test. PMN, polymorphonuclear neutrophils; SD, standard deviation; WBC, white blood cell. ESR, erythrocyte sedimentation rate;CRP, C-reactive protein.

### 3.2 mNGS and microbial culture results

**Figure 2:**
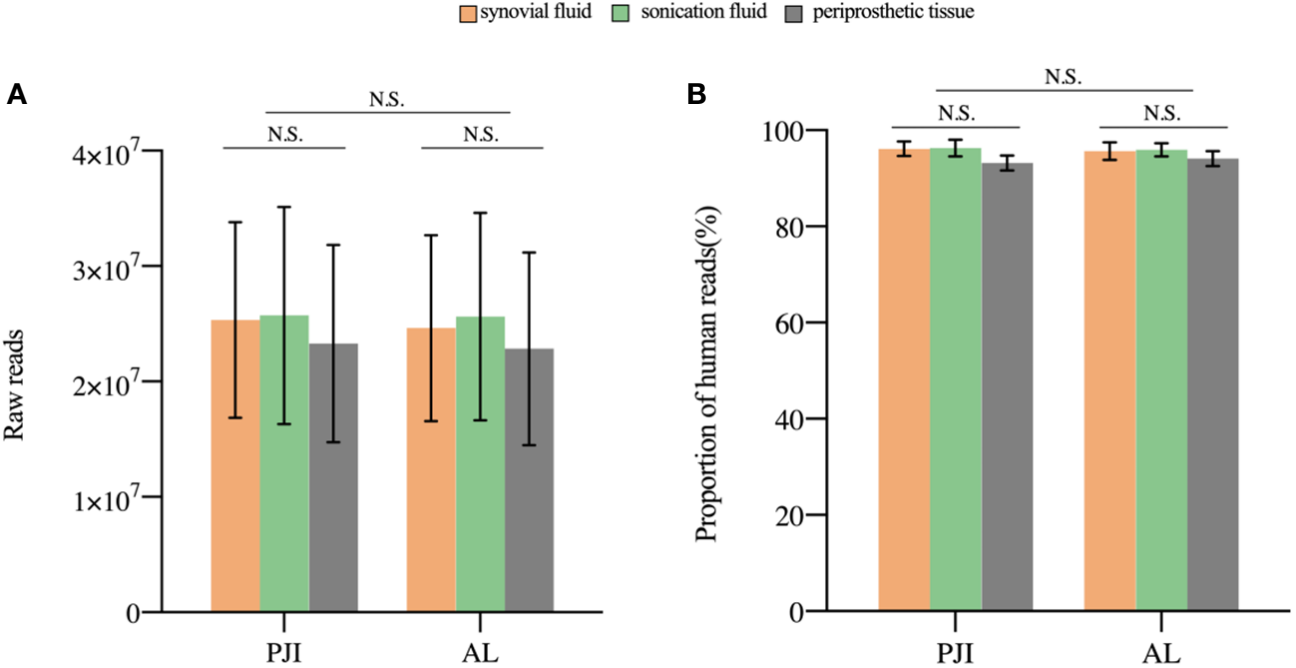
The sequencing characteristics of the different specimens. **(A)** The raw reads of the three samples in the PJI and AL groups was not statistically (*P*>0.05). **(B)** The difference in the proportion of human reads was not statistically significant (*P*>0.05). N.S., non-significant; PJI, periprosthetic joint infection; AL, aseptic failure.

A median 24,332,918 raw reads (interquartile range (IQR) 19,126,072 to 27,035,628) were generated from each sample sequenced. The human host nucleic acid sequences averaged 95.28% (ranging from 90.63% to 98.71%). Differences in raw reads and proportions of human nucleic acid sequences between synovial fluid, prosthetic sonication fluid, and periprosthetic tissue samples from the PJI and AL groups did not show statistical significance (*P*>0.05, **Figure 2**).

In the PJI group, 31 patients tested positive in culture, while 40 patients exhibited positive mNGS results. Within the AL group, one patient exhibited positive mNGS results, whereas all patients tested negative in culture.

The pathogens detected by both culture and mNGS are presented in **Supplementary Table 1**. Among the 43 PJI cases analyzed with culture, pathogenic microorganisms were isolated in 31 cases (72.1%), with all cases attributed to a single microorganism. Pathogenic microorganisms were isolated in 40 out of the 43 (93.0%) PJI cases using mNGS, 33 cases of infections were caused by a single microorganism, and 7 were polymicrobial.

**Figure 3:**
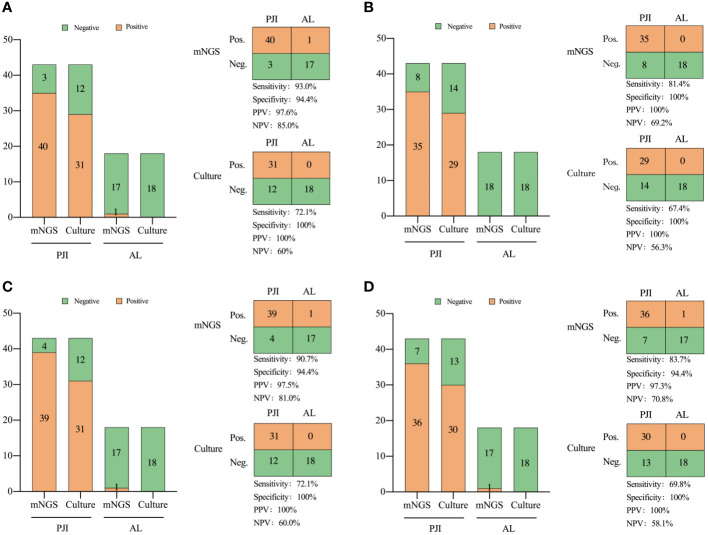
Comparison of the diagnostic efficiency of metagenomic next-generation sequencing and culture for PJI in: **(A)** all types of specimens, **(B)** periprosthetic tissue, **(C)** prosthetic sonication fluid, and **(D)** synovial fluid. NPV, negative predictive value; PPV, positive predictive value. Neg., negative; Pos., positive.

In the comparison of diagnostic performance between mNGS and culture, mNGS demonstrated higher sensitivity, both in the combined analysis of all specimen types and when considering each individual type of specimen. In summary, the sensitivity of mNGS was 93.0%, which was significantly higher than that of culture (72.1%, X^2^ = 6.541, *P*=0.011, **Figure 3**).

The turnaround time for mNGS in our patients was 24-28 hours. In contrast, the mean durations for pathogen culture feedback for bacteria, fungi, and mycobacteria were ≥3, 7, and 45 days, respectively. Thus, mNGS exhibits a time advantage in detection.

### 3.3 Consistency of mNGS and culture results

Comparing the mNGS and culture results among the 43 patients in the PJI group, 31 were double positive and 3 were double negative, while 9 showed positivity in mNGS but negativity in culture.

**Figure 4:**
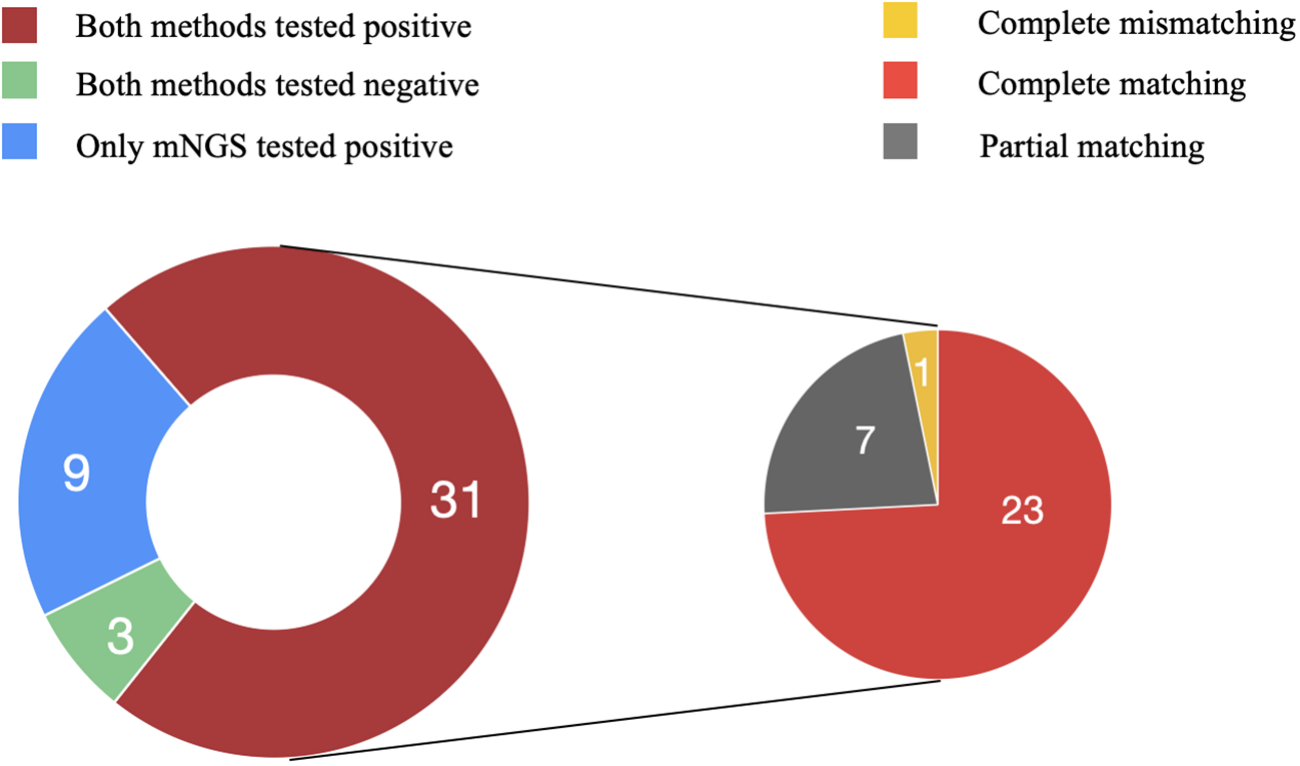
Consistency of metagenomic next-generation sequencing and culture results. Among the 43 patients in the PJI group, 31 were double positive and 3 were double negative, while 9 showed positivity in mNGS but negativity in culture. Among the double positive cases, 23 exhibited complete matching, 7 showed partial matching, and one case exhibited complete mismatching.

Among the double positive cases, 23 exhibited complete matching (*Staphylococcus aureus* in 10, *Staphylococcus epidermidis* in 5, *Staphylococcus haemolyticus* in 2, *Enterococcus faecalis* in 3, and *Escherichia coli* in 3), 7 showed partial matching (one of pathogens is the same). However, only one case exhibited a discordant result. Although *Enterobacter cloacae* was isolated by culture, the mNGS result indicated *S. epidermidis* as the pathogenic microorganism (**Figure 4**). The microbial results of mNGS and culture in PJI group are listed in **Supplementary Table 2**.

mNGS identified pathogenic microorganisms in 9 culture-negative cases, including *S. aureus* (2), *E. faecalis* (1), *S. haemolyticus* (1), *S. epidermidis* (1), *Mycobacterium abscessus* (1), *E. coli* (1), *E. cloacae* (1), *Candida albicans* (1). In the AL group, all culture results were negative. However, in one case, *E. coli* was detected in the sonication fluid by mNGS.

### 3.4 Consistency of mNGS for different types of specimens

The mNGS analysis of sonication fluid demonstrated a sensitivity of 90.7% and a specificity of 94.4%. The sensitivity of prosthetic sonication fluid mNGS was significantly higher than that of the synovial fluid and prosthetic tissues (*P* < 0.05).

Consistency in pathogen species detected by mNGS across the three specimen types demonstrates the reliability of mNGS and underscores its proficiency in detecting pathogens across varied specimen types. Among the results with pathogen matches, 26 cases exhibited identical positive sequencing, while 3 cases displayed identical negative sequencing. In the partially matched cases that most accurately reflected diagnostic capability, prosthetic sonication fluid (92.9%, 13/14) demonstrated the highest pathogen detection rate (both single and multiple pathogens), followed by synovial fluid (71.4%, 10/14).

### 3.5 Effect of antibiotic exposure on test results

**Figure 5:**
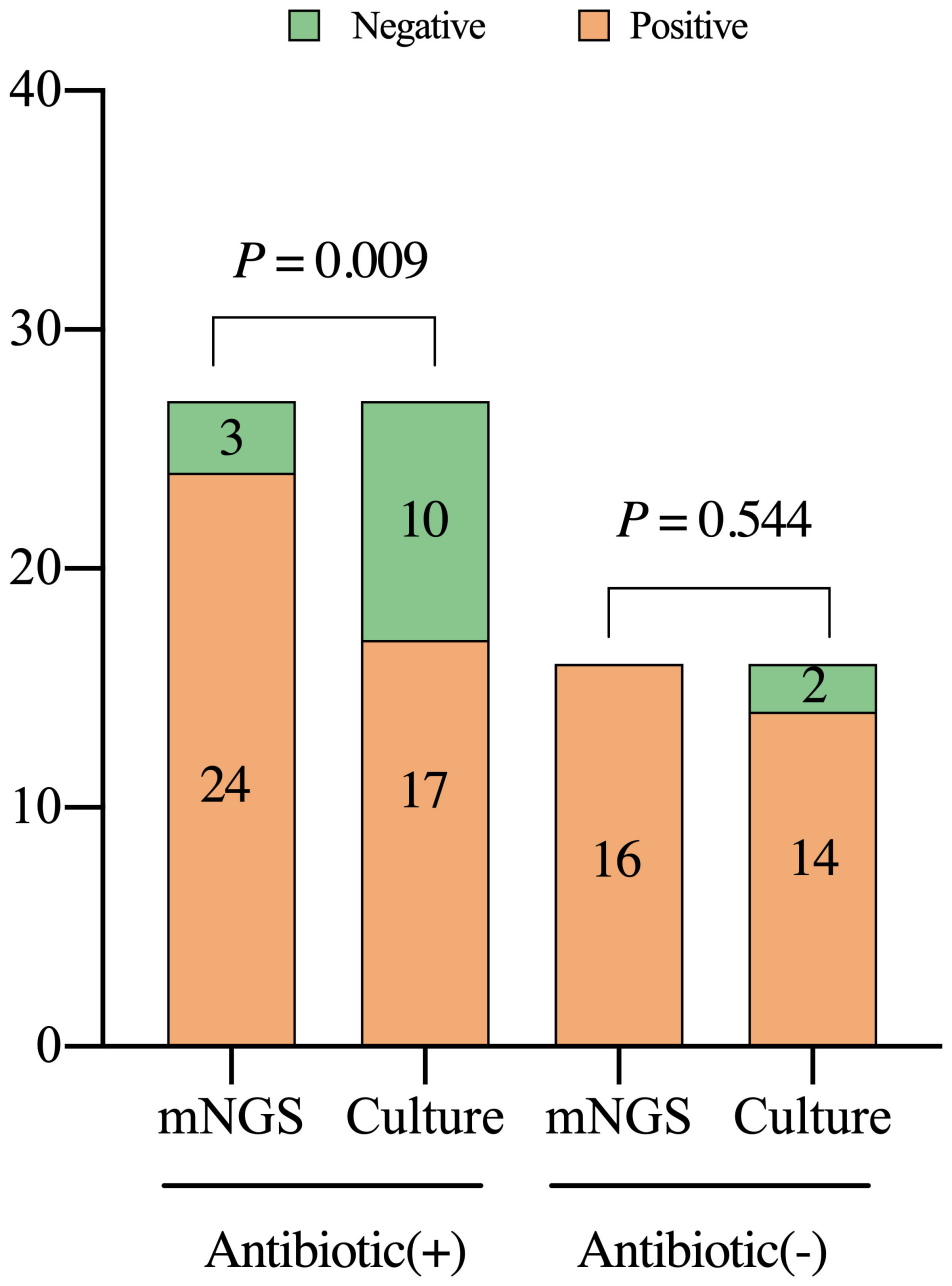
Detection characteristics of metagenomic next-generation sequencing and culture in periprosthetic joint infections among patients with or without previous treatment with antibiotics. Antibiotic (+), the patients were administered with antibiotics within two weeks prior to the test; mNGS, metagenomic next-generation sequencing.

The results of mNGS and culture are affected by antibiotic exposure. In the PJI group, mNGS demonstrated a sensitivity of 92.3% in 27 patients (62.8%) who applied antibiotics within 2 weeks before the test, which was significantly better than that of culture in 62.3%, (X^2^ = 6.857, *P*=0.009). Among the 16 patients not administered antibiotics, the sensitivity of the mNGS (93.8%) was not significantly different from that of the culture (87.5%) (X^2^ = 0.368, *P*=0.544, **Figure 5**).

### 3.6 Combined application of mNGS and culture

The combined application of mNGS and culture may enhance pathogen detection capabilities. Therefore, as shown in **Table 2**, we explored the effectiveness of the combined application of mNGS and culture based on the results when using multiple samples. The combination of mNGS and culture showed the highest sensitivity of 93.0% (40/43) across all three specimens. Prosthetic sonication fluid exhibited superior mNGS performance compared to other two types of specimen, making it the optimal choice for mNGS specimens in clinical practice. The combined application of mNGS using prosthetic sonication fluid and a single specimen culture achieved the highest sensitivity of 93.0% (40/43). Nevertheless, the performance difference between combining mNGS of prosthetic sonication fluid with culture and using mNGS of prosthetic sonication fluid only was not significant (90.7% vs. 93.0%, X^2^ = 0.156, *P* = 0.693).

**Table 2:** The combined application of mNGS and culture.

Detection combination method	Sensitivity, n (%)	Specificity, n (%)	PPV, n (%)	NPV, n (%)	Youden Index
mNGS (PT+SF+PSF) + Culture (PT+SF+PSF)	40/43 (93.0%)	17/18 (94.4%)	40/41 (97.6%)	17/20 (85.0%)	87.4
mNGS (PT+SF+PSF)	40/43 (93.0%)	17/18 (94.4%)	40/41 (97.6%)	17/20 (85.0%)	87.4
Culture (PT+SF+PSF)	31/43 (72.1%)	18/18 (100%)	31/31 (100%)	18/30 (60.0%)	72.1
mNGS (PSF) + Culture (PT+SF+PSF)	40/43 (93.0%)	17/18 (94.4%)	40/41 (97.6%)	17/20 (85.0%)	87.4
mNGS (PSF) + Culture (PSF)	40/43 (93.0%)	17/18 (94.4%)	40/41 (97.6%)	17/20 (85.0%)	87.4
mNGS (PSF) + Culture (PT)	39/43 (90.7%)	17/18 (94.4%)	39/40 (97.5%)	17/21 (80.9%)	85.1
mNGS (PSF) + Culture (SF)	39/43 (90.7%)	17/18 (94.4%)	39/40 (97.5%)	17/21 (80.9%)	85.1
mNGS (PSF)	39/43 (90.7%)	17/18 (94.4%)	39/40 (97.5%)	17/21 (80.9%)	85.1

mNGS, metagenomic next-generation sequencing; NPV, negative predictive value; PPV, positive predictive value; PSF, prosthetic sonicate fluid; PT, periprosthetic tissue; SF, synovial fluid; Youden Index = Sensitivity + Specificity − 1

## 4 Discussion

Accurate and rapid identification of infectious microorganisms in hip and knee PJI is crucial for guiding the application of antibiotics and improving infection control rate. At present, microbiological culture stands as the “gold standard” for diagnosing PJI. Nevertheless, the considerable false-negative rate of traditional culture presents several challenges in choosing antibiotics (Karbysheva et al., 2019). As a new molecular technology, mNGS sequences DNA fragments in clinical samples, extracting microbial sequence and species information through bioinformatics approaches (Adams et al., 2009). This study examined mNGS efficiency in detecting pathogens across three sample types using a multi-sample parallel control approach.

Our results show that the sensitivity of mNGS was higher than that of culture. These results align with our group’s prior statistical analysis studies on mNGS (Tan et al., 2022) and are consistent with studies by Yu et al. (2023) and Huang et al. (2020) on mNGS. Moreover, for culture-negative patients, mNGS showed outstanding detection ability (75.0%, 9/12). Thoendel et al. (2016) demonstrated that mNGS yielded positive results in 94.8% of culture-positive PJI cases. Moreover, in culture-negative PJI cases, it remained positive in 43.9% of instances. Ivy et al. (2018) demonstrated that in cases of PJI with negative microbial cultures, mNGS detected pathogenic microorganisms in 84% of cases. This is mainly due to the fact that mNGS combines high-throughput sequencing with bioinformatics analysis to directly detect nucleic acids extracted from samples and detect pathogenic bacteria without enrichment. This fundamentally avoids the problem of fastidious growth conditions for some pathogenic bacteria that are difficult to culture. In addition, among the double positive cases, 96.8% (30/31) were either completely matched or partly matched, with only one case exhibiting complete mismatching. The proportion of matched and partly matched cases among the double positive cases was > 90%, indicating an acceptable concordance between mNGS and culture.

The mNGS of prosthetic sonication fluid can detect more pathogenic microorganisms than synovial fluid and periprosthetic tissue. Trampuz et al. (2007) initially employed sonication to treat 120 joint prostheses and observed an enhanced detection rate of bacteria. In our study, mNGS could detect pathogenic microorganisms in the prosthetic sonication fluid of all 9 culture-negative PJI cases. Additionally, mNGS detected pathogens in 7 PJI cases involving multiple microorganisms. Thoendel et al. (2018) and Zhang et al. (2019) also showed that mNGS of prosthetic sonication fluid possesses high sensitivity in the detection of PJI pathogens. The detection rate of pathogenic microorganisms in the PJI group of our study surpassed that reported by Mei et al. (2023), who analyzed polymicrobial PJI cases using mNGS. Given its superior detection capabilities than synovial fluid and periprosthetic tissue, prosthetic sonication fluid stands out as the optimal choice for mNGS specimens. This is mainly attribute to the prosthetic sonication fluid can destroy the bacterial biofilm that colonizes the prosthesis, and enrich the concentration of pathogenic bacteria to be examined by centrifugation, which improves the detection efficiency of pathogenic bacteria. However, prosthetic sonication fluid also has its disadvantages, as it can only be obtained after intraoperative removal of the infected prosthesis, making it difficult to obtain a sample by puncture preoperatively to guide preoperative treatment (Huang et al., 2020).

The pathogenic bacteria detected by both methods in Sample 9 showed complete inconsistency. Several reasons could account for this inconsistency. Firstly, pitfalls in processing, establishment of background thresholds, and nucleic acid isolation can lead to failure to identify an organism and/or insufficient genome coverage to allow for accurate mapping to a species level. Secondly, the manifestation of PJI can be highly variable and the presence of other infecting organisms and the host microbiome can influence the relative abundances of various pathogens. Thirdly, insufficient DNA extraction can lead to a low number of reads or insufficient genome coverage in the setting of greater background reads. Weaver et al. (2019) similarly found discordance between genomical analysis and culture results was possible and reported positive cultures of organisms falling below their whole genome shotgun sequencing thresholds. Similarly, Ivy et al. (2018) observed that mNGS was incapable of identifying pathogens in 14 cases of culture-positive PJI.

While mNGS demonstrates distinct advantages in pathogen detection, its inherent limitations cannot be ignored. The mNGS results are susceptible to interference by background microorganisms, which mainly include exogenous bacterial and nucleic acid contamination caused by experimental manipulation or the experimental environment. Hence, rigorous adherence to sterilization and experimental protocols, implementation of blank controls, and rectification of false-positive results due to sample contamination are imperative. Moreover, the choice of the mNGS threshold significantly influences the outcomes. A threshold set too low could introduce numerous background microorganisms, causing false positives, whereas an excessively high threshold might overlook pathogenic bacteria, resulting in false negatives. The establishment of appropriate thresholds is a subject of controversy. Threshold settings must be carefully selected based on different sequencing methods and case types, ensuring a balance between diagnostic sensitivity and specificity. Generally, threshold setting relies on three indicators: the number of reads, RAG, and CR. In this study, we used a strict algorithm to obtain the results by reference to Street et al. (2017), employing a diagnostic threshold of RAG >15% and number of sequences reads > 100. In contrast to culture, mNGS presents challenges in conducting drug sensitivity tests for pathogenic bacteria, particularly for certain multi-drug resistant infections, thereby making it difficult to empirically administer antibiotics based on sensitivity test outcomes. In addition, the high cost of mNGS is a major barrier limiting its widespread use in the clinic practice.

There are some limitations in this study. Firstly, the variety of pathogenic bacteria detected in this study was limited, with some common pathogenic bacteria going undetected. This may be attributed to antibiotic use within the 2 weeks preceding testing, potentially hindering the growth of certain common microorganisms on the culture medium. Secondly, this study is a single-center study with a small sample size, which may also result in limitations in the types of pathogens detected and introduce bias into the results. To address these limitations, further support from larger samples, expanded datasets, and broader studies in subsequent stages is required. Finally, for multi-drug resistant infections, it is difficult for mNGS to conduct drug sensitivity testing and guide antibiotic selection. In a further study, we hope to conduct targeted detection of drug resistance genes to accurately guide the use of antibiotics.

## 5 Conclusions

In conclusion, mNGS can function as a precise diagnostic tool for identifying pathogens in PJI patients using three types of specimens. Due to its superior ability in pathogen identification, prosthetic sonicate fluid can replace synovial fluid and periprosthetic tissue as the optimal sample choice for mNGS. Additionally, employing three types of specimens in this study could contribute to the enhanced processing and analysis of mNGS technology, facilitating its thorough application in identifying pathogens in PJI patients.

## Data availability statement

The datasets presented in this study can be found in online repositories. The names of the repository/repositories and accession number(s) can be found below: China National GeneBank Database (CNGBdb) with accession number CNP0005321.

## Ethics statement

The studies involving humans were approved by Medical Ethics Committee of the First Affiliated Hospital of Zhengzhou University (Approval No:2020-KY-821). The studies were conducted in accordance with the local legislation and institutional requirements. The participants provided their written informed consent to participate in this study. Written informed consent was obtained from the individual(s) for the publication of any potentially identifiable images or data included in this article.

## Author contributions

JT: Writing – original draft, Writing – review & editing. LW: Writing – review & editing. LZ: Writing – review & editing. MS: Writing – review & editing. ZT: Writing – review & editing. JX: Writing – review & editing. HM: Writing – review & editing.

## Supplementary material

Supplementary Table 1 Detection of pathogens by mNGS and culture in the three types of specimen.

Supplementary Table 2 Microbial results of mNGS and culture in PJI group.
