# Supplementary material for: Optimal selection of specimens for metagenomic next-generation sequencing in diagnosing periprosthetic joint infections
Source: Front Cell Infect Microbiol. 2024 Mar 4;14:1356804. doi: 10.3389/fcimb.2024.1356804 (PMC10945027; doi:10.3389/fcimb.2024.1356804)
Supplement: Supplementary Table 1 — Detection of pathogens by mNGS and culture in the three types of specimen. [file DataSheet_1.docx]

**Supplementary Table 1.** Detection of pathogens by mNGS and culture in the three types of specimen.

| **Pathogens** | **No. of all detected microorganisms** | **Detected by mNGS** | | | **Detected by culture** | | |
| --- | --- | --- | --- | --- | --- | --- | --- |
|  |  | **SF** | **PT** | **PSF** | **SF** | **PT** | **PSF** |
| *Staphylococcus aureus* | 14 | 13 | 11 | 13 | 10 | 10 | 10 |
| *Enterococcus faecalis* | 5 | 5 | 4 | 5 | 3 | 2 | 3 |
| *Staphylococcus haemolyticus* | 4 | 3 | 2 | 4 | 2 | 2 | 2 |
| *Staphylococcus epidermidis* | 8 | 7 | 6 | 8 | 5 | 5 | 5 |
| *Mycobacterium abscessus* | 2 | 1 | 2 | 2 | 1 | 1 | 1 |
| *Escherichia coli* | 6 | 6 | 6 | 6 | 3 | 3 | 4 |
| *Enterobacter cloacae* | 3 | 2 | 2 | 3 | 2 | 2 | 2 |
| *Candida parapsilosis* | 1 | 1 | 1 | 1 | 1 | 1 | 1 |
| *Candida albicans* | 2 | 2 | 2 | 2 | 1 | 1 | 1 |
| *Mycoplasma hominis* | 1 | 1 | 1 | 1 | 1 | 1 | 1 |
| *Streptococcus agalactiae* | 1 | 1 | 1 | 1 | 1 | 1 | 1 |
| All | 47 | 42 | 38 | 46 | 30 | 29 | 31 |

mNGS, metagenomic next-generation sequencing; PSF, prosthetic sonicate fluid; PT, periprosthetic tissue; SF, synovial fluid.

**Supplementary Table 2.** Microbial Results of mNGS and Culture in PJI Group.

| **ID** | **Joint** | **Results of mNGS** | **Results of Culture** |
| --- | --- | --- | --- |
| 1 | Knee | *Staphylococcus aureus* | *Staphylococcus aureus* |
| 2 | Knee | *Staphylococcus aureus* | *Staphylococcus aureus* |
| 3 | Hip | *Staphylococcus epidermidis* | *Staphylococcus epidermidis* |
| 4 | Knee | *Enterococcus faecalis* | *Enterococcus faecalis* |
| 5 | Knee | *Staphylococcus aureus* | *Staphylococcus aureus* |
| 6 | Hip | *Escherichia coli* | *Escherichia coli* |
| 7 | Hip | *Staphylococcus aureus* | *Staphylococcus aureus* |
| 8 | Knee | *Staphylococcus aureus* | *Staphylococcus aureus* |
| 9 | Knee | *Staphylococcus epidermidis* | *Enterobacter cloacae* |
| 10 | Hip | *Staphylococcus epidermidis* | *Staphylococcus epidermidis* |
| 11 | Knee | *Mycobacterium abscessus*  *Staphylococcus haemolyticus* | *Mycobacterium abscessus* |
| 12 | Knee | *Staphylococcus aureus* | *Staphylococcus aureus* |
| 13 | Hip | *Enterococcus faecalis* | *Enterococcus faecalis* |
| 14 | Knee | *Candida parapsilosis*  *Enterococcus faecalis* | *Candida parapsilosis* |
| 15 | Knee | *Staphylococcus epidermidis* | *Staphylococcus epidermidis* |
| 16 | Knee | *Mycoplasma hominis*  *Enterobacter cloacae* | *Mycoplasma hominis* |
| 17 | Hip | *Staphylococcus aureus* | *Staphylococcus aureus* |
| 18 | Hip | *Staphylococcus aureus* | *Staphylococcus aureus* |
| 19 | Knee | *Enterobacter cloacae*  *Escherichia coli* | *Enterobacter cloacae* |
| 20 | Hip | *Staphylococcus haemolyticus* | *Staphylococcus haemolyticus* |
| 21 | Knee | *Staphylococcus epidermidis* | *Staphylococcus epidermidis* |
| 22 | Knee | Negative | Negative |
| 23 | Knee | *Escherichia coli* | *Escherichia coli* |
| 24 | Knee | *Enterococcus faecalis* | *Enterococcus faecalis* |
| 25 | Knee | *Enterococcus faecalis* | Negative |
| 26 | Hip | *Staphylococcus aureus* | Negative |
| 27 | Hip | *Staphylococcus haemolyticus* | *Staphylococcus haemolyticus* |
| 28 | Hip | Negative | Negative |
| 29 | Knee | *Escherichia coli*  *Staphylococcus aureus* | *Escherichia coli* |
| 30 | Knee | *Enterobacter cloacae* | Negative |
| 31 | Hip | *Staphylococcus aureus* | *Staphylococcus aureus* |
| 32 | Knee | *Staphylococcus aureus* | Negative |
| 33 | Knee | *Candida albicans*  *Staphylococcus aureus* | *Candida albicans* |
| 34 | Knee | *Mycobacterium abscessus* | Negative |
| 35 | Knee | *Candida albicans* | Negative |
| 36 | Hip | *Staphylococcus aureus* | *Staphylococcus aureus* |
| 37 | Hip | *Staphylococcus epidermidis* | Negative |
| 38 | Knee | *Escherichia coli* | Negative |
| 39 | Knee | *Escherichia coli* | *Escherichia coli* |
| 40 | Hip | *Streptococcus agalactiae*  *Staphylococcus epidermidis* | *Streptococcus agalactiae* |
| 41 | Hip | *Staphylococcus epidermidis* | *Staphylococcus epidermidis* |
| 42 | Hip | Negative | Negative |
| 43 | Knee | *Staphylococcus haemolyticus* | Negative |

mNGS, metagenomic next-generation sequencing

**Data availability statement**

The names of the repository/repositories and accession number(s) can be found below: China National GeneBank Database (CNGBdb) with accession number CNP0005321.
